# Vesicular Dysfunction and the Pathogenesis of Parkinson’s Disease: Clues From Genetic Studies

**DOI:** 10.3389/fnins.2019.01381

**Published:** 2020-01-08

**Authors:** Kirsten Ebanks, Patrick A. Lewis, Rina Bandopadhyay

**Affiliations:** ^1^Reta Lila Weston Institute, UCL Queen Square Institute of Neurology, University College London, London, United Kingdom; ^2^Department of Clinical and Motor Neuroscience, UCL Queen Square Institute of Neurology, University College London, London, United Kingdom; ^3^School of Pharmacy, University of Reading, Reading, United Kingdom; ^4^Department of Neurodegenerative Disease, UCL Queen Square Institute of Neurology, London, United Kingdom

**Keywords:** Parkinson’s disease, genetics, genome wide association studies, vesicular dysfunction, lysosomal dysfunction, alpha-synuclein, leucine-rich repeat kinase 2, Rab proteins

## Abstract

Parkinson’s disease (PD) is a common age-related neurodegenerative disorder with disabling motor symptoms and no available disease modifying treatment. The majority of the PD cases are of unknown etiology, with both genetics and environment playing important roles. Over the past 25 years, however, genetic analysis of patients with familial history of Parkinson’s and, latterly, genome wide association studies (GWAS) have provided significant advances in our understanding of the causes of the disease. These genetic insights have uncovered pathways that are affected in both genetic and sporadic forms of PD. These pathways involve oxidative stress, abnormal protein homeostasis, mitochondrial dysfunction, and lysosomal defects. In addition, newly identified PD genes and GWAS nominated genes point toward synaptic changes involving vesicles. This review will highlight the genes that contribute PD risk relating to intracellular vesicle trafficking and their functional consequences. There is still much to investigate on this newly identified and converging pathway of vesicular dynamics and PD, which will aid in better understanding and suggest novel therapeutic strategies for PD patients.

## Introduction

Parkinson’s Disease (PD) is a progressive and a debilitating neurodegenerative disorder which usually occurs in people in their sixth decade with an incidence of around 1% (De Lau and Breteler, 2006). The presenting clinical features at diagnosis include bradykinesia as an essential feature, together with resting tremor and rigidity (Gibb and Lees, 1988). Along with and occasionally preceding the motor symptoms are non-motor symptoms such as anosmia, constipation and sleep disturbances can also be observed (Schapira et al., 2017). With the progression of the disease, patients also develop non-motor features including dementia and neuropsychiatric symptoms (Politis et al., 2010). The loss of dopaminergic cells in the *substantia nigra* leading to a deficit of dopamine in the striatum is the cause of the typical motor features (Fearnley and Lees, 1991). Neuropathological characteristics include dopaminergic cell loss and the presence of Lewy bodies (LBs) and dystrophic neurites termed Lewy neurites (LNs) in the *substantia nigra* and other brain regions, the main component of which is fibrillar membrane bound forms of α-synuclein (Spillantini et al., 1997, 1998). The varied nature of the symptomology is reflected in the wide range of affected brain regions, with pathology spreading from the brainstem to the cortex (Braak et al., 2003). Notably, the LB pathology observed in PD is not restricted to this disorder, and are found in Alzheimer’s disease and also in asymptomatic individuals (also termed incidental LB cases) (Parkkinen et al., 2005). Outside of the central nervous system, LBs have also been described in peripheral nerve populations [reviewed in Surmeier and Sulzer (2013)].

Despite the initial clinical description of Parkinson’s syndrome more than two centuries ago, to date no disease modifying therapy has been approved for use in humans (Noyce and Bandopadhyay, 2017). Existing therapies are palliative in nature, with dopamine replacement as the main treatment strategy – an approach that does not halt or prevent disease progression. With regard to the underlying etiology, the majority of Parkinson’s cases are idiopathic with no discernible specific environmental or genetic cause, however, approximately 5–10% of cases are linked directly to deleterious inherited genetic variants (Reed et al., 2019). Over the past two decades mutations in at least 17 disease segregating genes have been identified [reviewed in Karimi-Moghadam et al. (2018)]. Recent Genome wide association studies (GWAS) have identified further loci across the human genome that are linked to increased lifetime risk for Parkinson’s in idiopathic disease (Kia et al., 2019; Nalls et al., 2019). Research into the actions and dysfunctions of the genes and their proteins have highlighted a number of common pathways in PD; affecting mitochondrial dysfunction, auto-lysosomal dysfunction, oxidative stress, vesicular dysfunction, and abnormal proteostasis (Zhou et al., 2008; Ebrahimi-Fakhari et al., 2012; Cieri et al., 2017). Additionally, PD is also influenced by non-cell-autonomous mechanisms such as cell-to cell transmission of protein aggregates (thought to be driven by a prion-like mechanism) and neuroinflammation (De Virgilio et al., 2016; Rey et al., 2018). In this review we will discuss our current understanding of vesicular dysfunction and abnormal protein handling and their role in the causation of PD, bringing together data from Mendelian forms of PD and GWAS nominated genes (Table 1).

**TABLE 1:** Table showing PD genes and GWAS hits discussed.

**Gene loci/Gene**	**Inheritance**	**Protein**	**Functions**
Park1/4 SNCA	AD	Alpha-synuclein	Vesicle fusion/autophagy
Park8/LRRK2	AD	Leucine-rich repeat kinase 2	Autophagy/endosomal functions
Park9/ATP13A2	AR	Cation-transporting ATPAse13A2	Lysosomes
Park17/VPS35	AD	Vacoular protein sorting 35	Endosomal functions
Park19/DNAJC6	AR	DNAJ subfamily C member 13	Endosomal functions
Park20/SYNJ1	AR	Synaptojanin1	Endosomal functions
Park21/DNAJC13	AD	DNAJ subfamily C member 6	Endosomal functions
Park23/VPS13C	AR	Vacoular protein sorting 13C	Endosomal functions
Unassigned/Rab39b		Ras like small GTPase	Related to alpha-synuclein
unassigned/GBA		Glucocerebrosidase	Lysosomal functions
*GAK*^∗^		*Cyclin-G associated kinase*	*Cellular adhesion and trans-golgi network*
*Rab7L1*^∗^		*Rab7L1*	*Links to LRRK2 to trans-golgi network and lysosomal trafficking system*
*Syt11*^∗^		*Synaptotagmin11*	*Autophagy, vesicle fusion*

*^∗^Denotes GWAS nominated genes. AD, autosomal dominant; AR, autosomal recessive.*

## Vesicular Mechanisms in PD Pathogenesis

**FIGURE 1:**
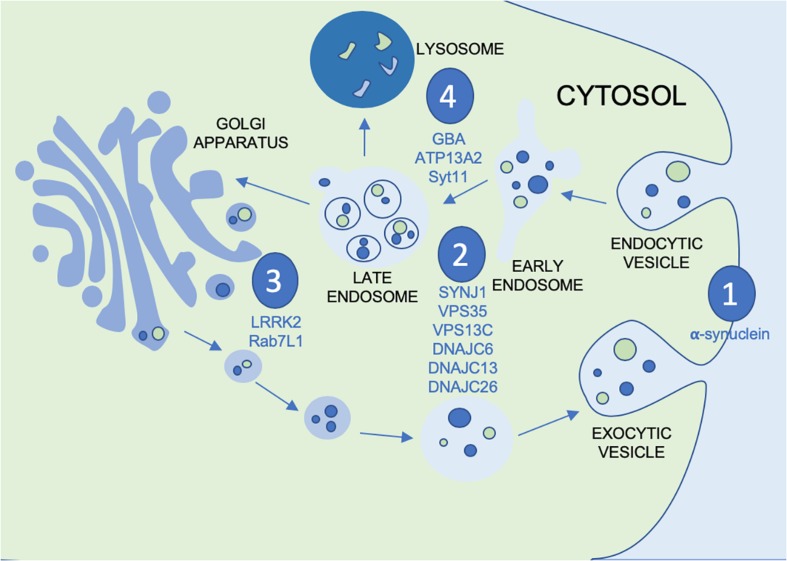
Figure depicting the vesicular process with the points of modulation of PD genes and GWAS nominated genes discussed in this review annotated by (1) vesicular fusion, (2) endocytosis, (3) *trans* golgi network and (4) lysosomes.

Vesicular mechanisms have been implicated in the pathogenesis of PD by way of several distinct pathways. Within the vesicular system there are several systems of interest, particularly as they apply to potential points of disease propagation and modulation. These points of interest can be seen in Figure 1: vesicular fusion (1), endocytosis (2), the *trans* golgi network (TGN) (3) and lysosomal functions (4). At each of these stages in the vesicular process genes have been identified to be familial linked and/or risk factors associated with PD. This not only provides viable evidence for the role of vesicular mechanisms in PD but also genes and proteins which can be investigated at each of these potential points of modulation along the vesicular network. At the point of vesicular fusion across membranes, α-synuclein has been implicated. Additionally, synaptojanin1 (SYNJ1), valosin containing proteins and DNAJC proteins have been shown to impact endocytic function. The *trans-*golgi network has also been shown to be impacted by leucine-rich repeat kinase 2 (LRRK2) and Rab proteins. Finally, glucocerebrosidase (GBA), ATP13A2 and synaptotagmin11 (SYT11) affect lysosomal functions. To better understand the possible impact this system may have on the progression of PD, the proteins acting at each point must be further investigated to better appreciate not only their role in the vesicular network, but also how this network can be modulated by therapeutic intervention or used as biomarkers for monitoring disease progression or treatment outcomes.

## Vesicular Fusion

### Alpha-Synuclein (α-Synuclein)

A missense mutation (the A53T transversion) in the α-synuclein (*SNCA*) gene was the first genetic variant to be unambiguously identified as causing PD (Polymeropoulos et al., 1997). Subsequently, further missense mutations (Kruger et al., 1998; Kiely et al., 2013; Zarranz et al., 2014) as well as duplications and triplications of the *SNCA* gene have been identified indicating that gene dosage is important for the pathogenesis of PD (Singleton et al., 2003; Ibanez et al., 2004). Additionally, polymorphisms in non-coding regions have been identified through GWAS as one of the risk factors for idiopathic PD (Simon-Sanchez et al., 2009) and an untranslated 3′ polymorphism increases α-synuclein expression (Soldner et al., 2016), however, how α-synuclein causes dopaminergic (DAergic) neuron degeneration remains unresolved.

Alpha-synuclein is a presynaptic protein which is relatively abundant in the brain (Maroteaux et al., 1988) and endogenous α-synuclein is necessary for DAergic neuron development (Garcia-Reitboeck et al., 2013). Electron microscopy has demonstrated α-synuclein in synaptic vesicles (Tao-Cheng, 2006) and has been shown to be associated with vesicles *in vitro* (Nakamura et al., 2008).

Alpha-synuclein is a natively unfolded protein, but adopts α-helical conformation in presence of membranes (Davidson et al., 1998; Bodner et al., 2009). Structural and biophysical analyses suggest that a membrane bound tetramer version of α-synuclein may represent the functional form of this protein (Bartels et al., 2011; Dettmer et al., 2013), although the physiological relevance of this remains a subject of debate (Fauvet et al., 2012; Theillet et al., 2016). One of the remarkable properties of α-synuclein is its ability to oligomerize, adopt a β pleated sheet conformation and aggregate under non-physiological conditions (Cho et al., 2009). Oligomerization of α-synuclein may contribute to its pathological function and can enhance its aggregation properties, with the majority of pathogenic coding variants increasing the propensity of α-synuclein to aggregate (Guan et al., 2016).

Importantly, aggregated α-synuclein is the most abundant component of Lewy bodies and dystrophic Lewy neurites in PD brain (Spillantini et al., 1998) and is also present in Dementia with Lewy bodies (DLB) brain and can sometimes be present in Alzheimer’s disease (AD) brain alongside AD pathology (Matej et al., 2019). Abnormal α-synuclein pathology is also present in oligodendroglial inclusions – termed glial cytoplasmic inclusions (GCIs) in Multiple system atrophy (Ahmed et al., 2012). A recent elegant study using super resolution microscopy has shown that Lewy pathology is interspersed with filaments, dysmorphic organelles together with membraneous structures and vesicles (Shahmoradian et al., 2019). This is important evidence of α-synuclein localization with vesicles and supports the hypothesis that compromised organelle trafficking is a putative driver of PD pathogenesis. These trafficking events could also be playing a part in prodromal PD, thus, making them important early stage points of α-synuclein modulation (Hunn et al., 2015).

A mechanistic link between α-synuclein and vesicular systems was noted as early as 1998 when it was found that, in the case of a familial (A30P) PD mutation (Kruger et al., 1998), the binding of α-synuclein to vesicles was inhibited (Jensen et al., 1998). This implicates the binding of α-synuclein to vesicular membranes in the clearance process providing a possible link between these mechanisms and the accumulation of α-synuclein. Furthermore, studies have shown that α-synuclein disrupts Golgi trafficking network (Cooper et al., 2006). It was also shown that this disruption could be rescued by Rab1, a known modulator of vesicular function (Cooper et al., 2006). Additionally, the reduction of α-synuclein in PD midbrain neurons was shown to restore previously inhibited lysosomal function (Mazzulli et al., 2016). Taken together, these data indicate a potential feedback relationship between α-synuclein and vesicular proteins. It may also provide evidence for the argument that the deposition of α-synuclein occurs further downstream than the disruption of vesicular and lysosomal functions implicating these mechanisms as the catalyst for a large portion of PD pathology.

Further evidence linking α-synuclein and vesicular dynamics was demonstrated by Burré et al., 2010), when oligomeric α-synuclein was shown to promote snare complexes both *in vivo* and *in vitro*, however, it was shown to inhibit membrane fusion. Moreover, binding of calcium to the c-terminus of α-synuclein augmented its lipid-binding property (Lautenschlager et al., 2018) and additionally α-synuclein overexpression facilitated its interaction with VAMP2 and modulate exocytosis (Sun et al., 2019) but whether this occurs in normal physiology remains to be determined. In addition, using cryoelectron tomography, Vargas et al. demonstrated that deletion of all three forms (α,β,γ) of synuclein increases synaptic vesicle (SV) tethering at the active zone but decreases the interlinking of SVs by short connectors (Vargas et al., 2017). It will be important to study how abnormal vesicular dynamics contribute to α-synuclein transition from physiological to abnormal forms which could be relevant for both normal physiology and PD pathology.

## Endocytosis

### Synaptojanin1 (SYNJ1)

Using homozygosity mapping and exome sequencing, a homozygous autosomal recessive mutation in *SYNJ1* gene was identified in a consanguineous family in Italy. The patients manifested Parkinsonism, dystonia motor features together with cognitive decline (Quadri et al., 2013). SYNJ1 encodes synaptojanin1, a phosphoinositide protein that plays important roles in synaptic vesicle endocytosis (Quadri et al., 2013). More recent studies have reported *R258Q* and *R459P* mutations in SYNJ1 associated with juvenile or early onset PD (Krebs et al., 2013; Kirola et al., 2016), and these mutations impair its phosphatase activity at a mechanistic level. By combining *SYNJ1* heterozygous mutation and LRRK2 *G2019S* mice impaired synaptic vesicle endocytosis was observed in midbrain neurons but not in cortical neurons, thus showing specific defects in dopamine containing neurons (Pan et al., 2017). Knockout of *SYNJ1* in mice results in impaired recycling of synaptic vesicles (Kim et al., 2002), and mice carrying a *SYNJ1* (*R258Q*) knock-in mutation present with striking endocytic defects associated with dystrophic axon nerve terminals were seen in the striatum (Cao et al., 2017). It will be important to test whether SYNJ1 related defects have a role in the pathogenesis of sporadic PD, and to clarify the details of its relationship with LRRK2 (Islam et al., 2016).

### Valosin Containing Proteins (VPS35 and VPS13C)

Through exome sequencing studies, two groups independently discovered heterozygous mutations in the *VPS35* gene linked with late-onset familial PD in 2011 (Vilariño-Güell et al., 2011; Zimprich et al., 2011). Although a number of coding variants have been reported to be associated with Parkinson’s, the one clear pathogenic mutation identified is the *D620N* variant, a relatively rare cause of autosomal dominant Parkinson’s affecting around 0.4% of all PD cases (Gambardella et al., 2016).

VPS35 is a component of retromer complex, which is involved in intracellular trafficking of proteins (Seaman et al., 1998) and is evolutionarily conserved (Zimprich et al., 2011). In rat cortical neurons, lentivirus-mediated gene transfer of *D620N* VPS35 mutation led to increased sensitization of neurons to several stressors; namely MPP+, rotenone and hydrogen peroxide and also increased cell death but no alteration in vesicular localization or abnormal retromer function was noted. Lentiviral overexpression of D620N mutation in dopaminergic neurons of the rat lead to neurodegeneration of substantia nigra and axonal damage whereas WT-VPS35 overexpression produced an intermediate level of neuropathology (Tsika et al., 2014).

A metanalysis of gene expression data from post mortem samples has shown significant downregulation of VPS35 mRNA levels in PD compared to controls (MacLeod et al., 2013), which was also observed in laser-microdissected PD SN dopamine neurons. Although whether these are reflected at protein levels remains uncertain (Tsika et al., 2014). Further protein level studies with large number of PD and control cases should be a priority. A key caveat to our understanding of how *VPS35* mutations link to Parkinson’s is that, to date, no VPS35 mutation brains have come to post-mortem pathological analysis, and therefore it is not known if *VPS35* PD is a synucleinopathy. It is of interest to note that VPS35 (D620N) mutation causes tau pathology in mice (Chen et al., 2019) and its levels are reduced in two primary tauopathies progressive supranuclear palsy and Pick’s disease (Vagnozzi et al., 2019).

An recent study by Mir et al. (Mir et al., 2018) demonstrated that VPS35 has an upstream role regulating LRRK2s kinase activity in phosphorylating Rab10. Therefore, modulating VPS35 actions as a mechanism to supress LRRK2 activity could be another approach to a disease modifying strategy in addition to LRRK2 inhibitors, which are already in Phase1 clinical trial (Hatcher et al., 2017).

VPS13C is the second member of the VPS family that has been linked to the pathogenesis of PD. Allelic variations which leads to premature termination of VPS13C cause autosomal recessive early onset PD. At post-mortem these cases harbored LBs in the *substantia nigra*, as well as in extra-nigral regions (Lesage et al., 2016). Using yeast models, VPS13 has been shown to function in the TGN-endosomal cycle in combination with the calcium binding protein Cdc13 (De et al., 2017). The same study (De et al., 2017) also linked VPS13C with lipid membranes. Lesage et al. demonstrated reducing VPS13C in cells using siRNA resulted in abnormal mitochondrial respiratory rates and also exacerbated Parkin-dependent mitophagy (Lesage et al., 2016). More recently Kumar et al. (Kumar et al., 2018) noted VPS13-linked PD mutations links to lipid transport between organelles and endoplasmic reticulum and this can lead to defects in membrane lipid homeostasis. These functions could directly or indirectly associate with vesicular functions and proteostasis dysfunctions in the context of PD pathogenesis.

### DNAJC6, DNAJC13, and DNAJC26

DNAJC proteins are part of the heat shock family of proteins which predominantly play a role in stress response (Pauli et al., 1992; Piano et al., 2004). Given this role as well as their action within the vesicular pathway, their possible involvement in disease has been explored as it relates to the regulation of stress response. Mutations in these genes have been shown to result in disease pathogenesis by way of disrupted protein folding and degradation processes, impaired endosomal transport and vesicular fusion, and the dysfunction of clathrin-mediated trafficking (Roosen et al., 2019).

The two DNAJCs with the most direct link to PD are DNAJC6 (also called auxilin1) and DNAJC26 (also known as GAKor auxilin2, a GWAS nominated gene). Both have been shown to play a role in clathrin-mediated trafficking and have been found to be either known sites of deleterious mutations or risk factors for PD by GWAS (Edvardson et al., 2012; Nalls et al., 2014). Auxilin, which plays a role in endocytosis, has been found to be associated with juvenile onset Parkinsonism (Edvardson et al., 2012; Köroğlu et al., 2013; Elsayed et al., 2016; Nguyen and Krainc, 2018). One possible mechanism through which this occurs was found to be disruption of clathrin-mediated synaptic vesicle endocytosis by LRRK2 phosphorylation of auxilin in DA neurons (Nguyen and Krainc, 2018). Similarly, GAK, a serine/threonine kinase which is structurally analogous to auxilin, has been shown to be involved at the synaptic vesicle membrane level in experimental systems (Kanaoka et al., 1997; Nagle et al., 2016). This however, needs to be validated *in vivo*. Once again, clathrin-mediated vesicle binding is implicated as the mechanism of dysfunction (Nagle et al., 2016). Interestingly, the same study also found a potential link to mitochondrial function, which, given GAK’s previously described mechanism of action may indicate a possible role of mitophagy in GAK mediated PD risk. GAK was identified as a putative risk factor locus by a large scale meta-analysis (Nalls et al., 2014). Its expression is ubiquitous, however, lower amounts are expressed in the brain (Roosen et al., 2019). A substrate of GAK phosphorylation is ATP1a3 as demonstrated by chemical genetic identification (Lin et al., 2018) and this is necessary for cargo trafficking. Additionally, GAK has been shown to phosphorylate clathrin heavy chain and AP2 which play a role in vesicle membrane formation and cargo packaging at the cell membrane, respectively, adding further evidence to the role of GAK in the vesicular process (Korolchuk and Banting, 2002; Yabuno et al., 2019). In contrast to DNAJC6/auxilin, GAK is an essential protein during development and adulthood in mice, with GAK knockout being lethal (Lee et al., 2008). It is clearly important to study in more detail the interactions of all the PD risk factors at the level of vesicular functions.

DNAJC13 has also been linked to endosomal function (Girard et al., 2005; Fujibayashi et al., 2007). While cases of PD caused by DNAJC13 mutations are relatively rare, several mutations of the gene have been linked to disease (Gustavsson et al., 2015; Lorenzo-Betancor et al., 2015; Rajput et al., 2015; Ross et al., 2016). After familial cases segregating with DNAJC13 mutations were found, further investigation into the mechanisms of action yielded results which implicated endosomal dysfunction in disease pathogenesis (Vilariño-Güell et al., 2013). The mechanism through which this has been speculated to happen involves the interaction of DNAJC13 with retromer (Popoff et al., 2009). Retromer deals with the targeting and directing of specific endosomes to the *trans-*Golgi network for recycling (Bonifacino and Hurley, 2008), and provides a link to VPS35 (see above) and a number of other Parkinson’s related genes. This implicates DNAJC13 in the regulation of vesicle sorting and thus dysfunction of this protein linked to disease may result in improper sorting of endosomal cargo.

DNAJCs have been implicated in a range or complex neurodegenerative disorders, many of which have a parkinsonian component. Much like the previously discussed proteins, DNAJCs could be potential therapeutic targets for PD. Given the role they play in vesicle docking and fusion they could be an important point of modulation to increase movement of vesicular cargo across membranes in the presence of pathology. However, the protein-protein interactions of many of the DANJCs may pose challenges for therapeutic targets with GAK being the possible exception, given its role as a kinase making it a more tractable candidate.

## *Trans* Golgi Network

### LRRK2 (Leucine-Rich-Repeat Kinase 2)

Autosomal dominant LRRK2 mutations were identified in 2008 by two groups (Paisán-Ruíz et al., 2004; Zimprich et al., 2004), with mutations in this gene now recognized as one of the most common genetic contributors to heightened risk of PD affecting different ethnic populations variably, and has also been implicated in sporadic PD (Lesage et al., 2006; Ozelius et al., 2006; Healy et al., 2008; Nalls et al., 2014). The protein product of LRRK2 is a 2527 amino acid/286kDa scaffolding protein with multiple independently acting domains (Gotthardt et al., 2008). LRRK2 protein has ubiquitous expression and is increased in microglia following inflammatory stimuli in mice (Moehle et al., 2012). The large size of the protein and the number of distinct domain structure of the LRRK2 molecule is, however, consistent with the number of functions and the various protein domains is suspected to serve with functions including but not limited to, vesicular trafficking, autophagy and immune response (Rideout and Stefanis, 2014; Wallings et al., 2015). Crucially, LRRK2 associates with membranous structures and vesicles in mammalian brains and recently, integrated-omics analysis identified dysregulation of endocytic pathway in iPS derived DAergic neurons carrying G2019S mutation in the LRRK2 gene (Connor-Robson et al., 2019).

As a member of the ROCO super family, LRRK2 consists of a Ras of complex domain (ROC)/GTPase domain and a kinase domain (KIN) linked by the carboxyl terminal of ROC (COR) (Li et al., 2014). The two active enzymatic domains are at the heart of LRRK2 cellular function, with pathogenic coding variants altering kinase and GTPase function (Alessi and Sammler, 2018). The two enzymatic domains are flanked on either side by several protein-protein interacting domains. Interestingly all of the pathogenic LRRK2 mutations are within the GTPase and kinase domains and they affect the kinase activity. This makes LRRK2 a potentially druggable target, and indeed LRRK2 kinase inhibitors are currently undergoing human trials (Alessi and Sammler, 2018).

Research into the LRRK2 interactome has implicated LRRK2 in cellular trafficking and transport via a number of direct interactors and effector proteins (Manzoni et al., 2015). The link between LRRK2 and lysosomes has been highlighted by the identification of a number of Rab GTPases as substrates for the kinase activity of LRRK2 by proteomic analyses (Steger et al., 2016; Eguchi et al., 2018). Additionally, in a screen for LRRK2 interactors revealed specifically cyclin-G associated kinase (GAK) and Rab7L1/Rab29, a Ras related protein which deals primarily with membrane trafficking, as direct interactors of LRRK2 (Beilina et al., 2014). Rab7L1 was found to be phosphorylated by LRRK2 and is involved in vesicular clearance through the *trans-*Golgi network (Liu et al., 2017). It was shown that LRRK2, phospho-Rab 8/10 together with Rab7L1 help to maintain homeostasis in stressed lysosomes (Eguchi et al., 2018; Jeong et al., 2018) and phosphomutants of Rab proteins at conserved LRRK2 phosphorylation sites induces neurotoxicity and dopaminergic neuron degeneration in mice (Jeong et al., 2018). In human iPS neurons, in wild-type and LRRK2 mutated neurons, it was shown that LRRK2 may be playing a role by phosphorylating auxilin in its clathrin domain at Ser627 which is abolished upon kinase inhibition (Nguyen and Krainc, 2018), and transmission electron microscopy showed decreased synaptic vesicle density in presynaptic nerve terminals of R1441C dopaminergic neurons indicating defective synaptic vesicle endocytosis (Nguyen and Krainc, 2018). This is linked to the accumulation of oxidized dopamine in mutant neurons leading to decreased GBA activity and accrual of α-synuclein (Nguyen and Krainc, 2018). However, the phosphorylation of auxilin by LRRK2 needs to be verified endogenously using site specific phospho-antibody. LRRK2 has also been shown to be linked to the lysosomal pathway and autophagy (Manzoni et al., 2013a) and LRRK2 may phosphorylate EndophilinA to promote autophagy, however, this has not been shown in an endogenous context (Manzoni et al., 2013b; Soukup and Verstreken, 2017). Whilst all these studies provide more compelling evidence for the role of LRRK2 in PD, a key emerging theme of interest is the common mechanism of these LRRK2 interactors. These data tentatively link LRRK2 and vesicular mechanisms although, given the multitude of interactors LRRK2 has been linked to Manzoni et al. (2015) it is difficult to exclude other cellular pathways as being important for disease pathogenesis in LRRK2 mutation cases.

A number of studies highlight a complex link between α-synuclein and LRRK2, and how they might interact in the pathogenesis of PD. It has been shown that LRRK2 alone cannot induce Parkinsonism in a transgenic mouse model but when overexpressed in an α-synuclein transgenic model it does appear to contribute to the acceleration of the disease (Lin et al., 2009). Furthermore, ablation of LRRK2 in this model does appear to offer protection from neurodegeneration to some degree (Lin et al., 2009; Tsika et al., 2014). However, expression of human *R1441CLRRK2* in mice DAergic neurons failed to cause neurodegeneration nor did the mice accumulate abnormal protein inclusions (Tsika et al., 2014). A recent study by Hendersen et al. provides evidence that this may partly derive from LRRK2 acting to potentiate cell to cell transmission of α-synuclein aggregates, however, it should be noted that not all LRRK2 cases present with the aforementioned aggregates lending to pleiotropic pathology in LRRK2 cases (Henderson et al., 2019; Lewis, 2019). This suggests that there is interplay between α-synuclein and LRRK2, however, the mechanisms through which they interact have remained largely elusive. Additionally, G2019S knock-in mice showed progressive dysfunction of dopamine transporter along with serine-129 phosphorylated α-synuclein accumulation at 12 months of age (Longo et al., 2017). It is possible that the missing functional link may be through vesicular transport wherein α-synuclein and LRRK2 have a dependent relationship as a result of their shared interactions with vesicular proteins.

### Rab Proteins

Rab GTPases are members of the Ras superfamily which regulate vesicular mechanisms. These proteins have been shown to play a part in various neurodegenerative diseases through their role in cellular functions including but not limited to endocytosis, vesicle trafficking, vesicle docking, ciliogenesis and interactions in the *trans-*Golgi network (Schimmöller et al., 1998; Rodman and Wandinger-Ness, 2000; Seabra et al., 2002). While the Rab family encompasses roughly 50 proteins with variable functions across the vesicular pathway, 10 have been shown to have a possible relationship to PD either directly or indirectly (Gonçalves et al., 2016; Steger et al., 2016; Chung et al., 2017). The myriad of functions attributed to the Rab proteins and the varied hypothesized routs to pathogenesis in PD can be summarized as the following three pathways: endocytic function and lysosomal stress, ciliogenesis and sonic hedgehog signaling and, lastly the *trans-*Golgi network and endoplasmic reticulum (ER) stress.

With regard to direct evidence of a link between Rab proteins and PD, genetic analysis of three male members of an Australian family with early onset parkinsonism and learning disability revealed a 45 Kb deletion in the Rab39B gene located on the X chromosome, resulting in a complete loss of the protein. In another unrelated Wisconsin kindred, a loss of function missense mutation (c.503C > A) was identified (Wilson et al., 2014). This mutation completely destabilized the protein mimicking a loss of function. Post-mortem analysis of the brain demonstrated widespread LB accumulation along with extensive dopaminergic cell loss in the *substantia nigra*. Iron accumulation, tau immunoreactivity and axonal spheroids were also noted (Wilson et al., 2014). Further to this study, Mata et al. described another X-linked dominant mutation (p.G192R) in the Rab39B gene with reduced penetrance seen in females (Mata et al., 2015). Transient expression of the mutant protein in immortalized cells resulted in its mislocalization (Mata et al., 2015). It is important to note that RAB39b is neuron specific and plays a role in synapse formation and maintenance (Giannandrea et al., 2010). A ShRNA-based screen identified several Rab proteins from endocytic recycling pathway acted as genetic modifiers of α-synuclein secretion, aggregation and toxicity (Gonçalves et al., 2016). Examination of Rab proteins and vesicle recycling components in the context of PD are warranted which might open up novel avenues for therapeutic intervention. While the role of Rab39B in vesicular trafficking is less clear, it has been shown to play a role in α-synuclein clearance and studies have shown that mutations in this gene can result in PD-like symptoms, intellectual deficits and even early onset PD (Wilson et al., 2014). This can be taken together as a possible pathway to PD through a dysfunction in endosomal processes leading to lysosomal stress, reduced clearance of α-synuclein and ultimately increased α-synuclein deposition.

A number of Rab proteins (RAb8b, Rab11a, and Rab13) have impact on α-synuclein clearance which can have consequences on α-synuclein aggregation (Gonçalves et al., 2016). Similarly, Rab8A has also been shown to play a role in membrane trafficking and clearance as well as protein transport (Chung et al., 2017). Further evidence for the role of Rab8A in development and pathogenesis comes as studies have shown that LRRK2 phosphorylation of Rab8A can cause centrosomal defects and thus cause widespread effects on neurite growth and migration (Madero-Pérez et al., 2018a).

Additionally, Rab proteins are associated in cilia formation and ciliogenesis is implicated in PD pathogenesis. In this regard, LRRK2 R1441C mice show defects in cilia in their brains and LRRK2 kinase activity can block primary cilia formation in cultured cells with phosphorylation of Rab10 acting as a negative regulator in this context (Dhekne et al., 2018). Ciliary signaling is pertinent in olfactory functions (Dhekne et al., 2018) and anosmia is one of the first symptoms of PD. Holistically, this paints a compelling picture of the role of Rabs in neuronal development and how disruption of these pathways both in development and adulthood may lead to PD pathogenesis.

Lastly, disruption of the ER and *trans-*Golgi network have also been found to lead to PD- like pathology. Rab7L1 (also called Rab29) is not only another LRRK2 interactor but also a candidate for the PARK16 locus (Nalls et al., 2014; Steger et al., 2016). Further exploration of the interaction of LRRK2 and Rab7L1 in PD implies that their joint function has primarily to do with sorting through both the Golgi apparatus and lysosomal systems (MacLeod et al., 2013). One possible mechanism for this dysfunction is a change in Golgi morphology mediated by LRRK2 phosphorylation of Rab7L1 (Fujimoto et al., 2018). Furthermore, Rab7L1 promotes recruitment of LRRK2 to the *trans-*Golgi network as well as LRRK2 autophosphorylation thus implicating a malignant feedback relationship between LRRK2 and Rab7L1 in the presence of pathogenic LRRK2 mutations (Liu et al., 2017). Rab7L1 has also been shown to play a role in lysosomal homeostasis along with the aforementioned Rab8A and Rab10 (Eguchi et al., 2018). It was proposed that the recruitment of these Rabs onto enlarged lysosomes ultimately promoted lysosomal secretion and inhibited further enlargement (Eguchi et al., 2018). This stabilizing function was mediated by the recruitment of LRRK2 and Rab7L1 from the Golgi onto the stressed lysosomes placing Rab7L1 further upstream on this pathway than Rab8A and Rab10 even as they serve a similar regulatory function (Eguchi et al., 2018). This is further confirmed by the finding that the centrosomal deficits caused by Rab8A phosphorylation by LRRK2 as it was recruited on to the Golgi by Rab7L1 (Madero-Pérez et al., 2018b). This taken together would indicate that relocalization of LRRK2 mediated by Rab7L1 may result in many of the previously discussed deleterious interactions with Rabs and other yet unknown effectors further downstream. Studies to unveil the mechanistic details of Rab protein functioning and its downstream consequences as seen by its interaction with RIPL1 (Dhekne et al., 2018) could open new therapeutic avenues for PD treatment.

## Lysosomal Function

### GBA (Glucocerebrosidase)

Gaucher’s disease (GD), inherited in autosomal recessive pattern results from the deficiency of the enzyme GBA and is the most common lysosomal storage disease (Tsuji et al., 1987; Hruska et al., 2008). In addition to multisystem lysosomal storage dysfunction, a proportion of patients with neuropathic GD presenting with the clinical features of PD, and GD associated mutations in the heterozygous state act as a strong risk factor for idiopathic PD (Sidransky et al., 2009), and to date more than 300 GBA mutations have been reported. In addition, GWA studies have identified GBA variants as risk factors of PD (Simon-Sanchez et al., 2009; Blauwendraat et al., 2018). In normal physiology, GBA cleaves off glucose for use in lysosomal metabolic processes, thus, the dysfunction of this enzyme can lead to overall lysosomal dysfunction (Do et al., 2019). Crucially, GBA-PD and sporadic PD patients show a loss in GBA activity and GBA levels (Gegg et al., 2012; Murphy et al., 2014).

Sardi et al. (Sardi et al., 2011) provided evidence that a mouse model of GBA (D409V/D409V) showed characteristics of synucleinopathies including progressive accumulation of proteinase K resistant α-synuclein accumulation and cognitive deficits. Additionally, exogenous administration using adeno associated virus containing recombinant GBA can overcome pathological features in mice (Sardi et al., 2011). In another study, Glucosylceramide (GlcCer), the GBA substrate, control the formation of α-synuclein assemblies in primary neurons and in human iPS neurons leading to neurodegeneration (Mazzulli et al., 2011). They also suggested a bidirectional process between accumulation of toxic α-synuclein species and GCase levels which may lead to self-propagation of disease (Mazzulli et al., 2011). GBA1 KO mouse embryonic fibroblasts and in-patient derived fibroblasts with GBA1 mutation affects lysosomal recycling resulting in Rab7 accumulation in lysosomes (Magalhaes et al., 2016) while GBA KO in drosophila resulted in abnormal lysosomal pathology, mTOR activity was downregulated and rapamycin ameliorated the lifespan of flies (Bai et al., 2015; Kinghorn et al., 2016). It is important to note that all of the GBA cases that have been examined at post-mortem harbor α-synuclein pathology (Schneider and Alcalay, 2017).

### ATP13A2 and Synaptotagmin 11 (SYT11)

Autosomal recessive mutations in ATP13A2 cause an early onset parkinsonian syndrome (kufor rakeb syndrome, KRS) and have also been linked to neuronal ceroid lipofuscinosis (Ramirez et al., 2006; Crosiers et al., 2011). It belongs to the 5P-type subfamily of ATPase and is a lysosomal transmembrane protein (Ramirez et al., 2006). Loss of ATP13A2 leads to instability of the lysosomal membrane leading to impaired lysosomal function and proteolysis which could result from imbalance of divalent cation levels (Dehay et al., 2012; Tofaris, 2012). Evidence from one brain studied at autopsy with mutation in ATP13A2, has shown absence of LBs but presence of lipofuscinosis in cortex, basal nuclei and cerebellum and retina (Bras et al., 2012). One possibility is that that the disruption of divalent cation homeostasis within neurons could be responsible for lysosomal defects and neurodegeneration, however, the precise mechanisms underpinning disease are, to date, unclear (Usenovic et al., 2012; Lubbe et al., 2016).

Synaptotagmin 11 (SYT11), also a GWAS nominated gene, is a protein which deals largely with vesicular fusion with the membrane and exocytosis (Südhof, 2012; Nalls et al., 2014). Much like ATP13A2, SYT11 regulates autophagy by way of modulating lysosomal function (Bento et al., 2016). It has been found that not only do decreased levels of SYT11 induce lysosomal dysfunction but it is required for parkin-linked dysfunction in PD (Bento et al., 2016; Wang et al., 2018). Furthermore, a recent study has affirmed the relevance of the originally found GWA locus for GBA, the GBA-SYT11 locus, implicating a link between SYT11 and other lysosomal PD genes such as GBA (Blauwendraat et al., 2018).

## PD as a Dysfunction of Vesicular Mechanisms

**FIGURE 2:**
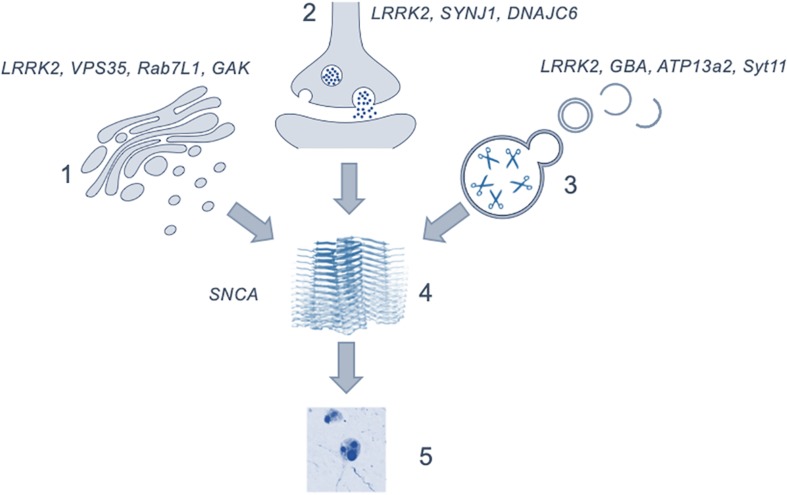
Figure depicting the possible role of the discussed mechanisms as upstream effectors in the generation of α-synuclein aggregation and Lewy body formation. (1) showing the genes involved in the *trans-*golgi network; (2) in endocytosis and exocytosis; (3) lysosomal genes and (4) the generation of α-synuclein oligomers leading to the formation of (5) Lewy bodies. The precise point at which cytotoxicity occurs in the brain is not clear. Image of α-synuclein fibrils (4) is modified from Li et al. (2018) using a Creative Commons license.

The regulation of intracellular vesicles formation and trafficking is central to cellular function, with disruption of the pathways involved in these processes having serious consequences for health. Evidence from human genetics and model systems support a key role for vesicular biology in the etiology of PD, stretching across a range of organelles and cell types. Given the complex interactions between vesicular proteins and the role of multiple pathways and networks in maintaining vesicular homeostasis, it is becoming clear that disruption at a number of points in vesicle regulation in the human brain can result in neurodegeneration and the development of parkinsonism. Importantly this implies that many of the genes, protein interactions and pathways discussed in this review may be valid pathological origins for PD and is summarized in Figure 2. The range of cellular events impacted by vesicle trafficking, coupled with the array of cell types and brain regions involved, could account for the wide variety of possible genetic offenders and the variability of disease profile within patient cohorts. Studies have begun to focus on this more dynamic perspective, describing the pathological presentation in terms of affected pathways rather than singular genes and proteins as what would amount to organelles crowded with non-functioning vesicles and vesicular proteins (Shahmoradian et al., 2018). It is clear that to use these insights into vesicular trafficking and the pathogenesis of Parkinson’s in a meaningful way to inform the development of novel therapeutics, the proteins which work within these pathways must be better understood and, furthermore, the mechanisms by which they interact with one another must be explored.

As seen by the graphical representation of vesicular system shown in Figure 1, there are many possible points of systemic deficit but also many points of potential modulation. To tease out these possible therapeutic targets an expansion of the interactors of each these proteins as well as their isomeric forms would be necessary. Given that a large portion of what would appear to be the targets further upstream of α-synuclein, such as the previously discussed Rab7L1, GAK, and ATP13A2, are most functionally active along the golgi- lysosomal axis further exploration of their interactors and regulators would clarify the role of this system in pathogenesis.

## Conclusion and Future Perspective

Our understanding of the genetic landscape of PD has expanded rapidly over the past two decades through increased identification of Mendelian inheritance genes and the nomination of risk loci through GWA studies. The challenge for researchers is to translate the risk genes identified using GWAS, integrate these with Mendelian genes and move toward unraveling novel pathways for disease modification treatments. Some groups have already begun deciphering the points of interaction of some of the risk factors as being at the synapses (Nguyen and Krainc, 2018; Wang et al., 2018) which could well be a starting point of disease pathogenesis and could occur long before clinical symptoms appear. Based upon these advances, our understanding of the disease processes that contribute to PD is maturing. Further studies involving other risk genes are highly warranted at this time point.

Clues from neuropathology studies suggest that PD is a multifactorial disorder, with α-synuclein spread occurring in a systematic way from one brain region to another which are neuroanatomically connected (Braak et al., 2003). The prion-like propagation of α-synuclein pathology may be an important event in disease pathogenesis (Luk et al., 2012; Peng et al., 2018). A systematic approach is now needed to weave together the potential risk factors involving the entire vesicular pathway and ascertain a temporal sequence of events. Importantly, the key will be in understanding how these factors contribute to aggregation of α-synuclein, the major pathological player in sporadic PD and also in several of the Mendelian forms of the disease (Schneider and Alcalay, 2017). Such studies should also foster discoveries of early diagnostic biomarker tools, which remains another critical unmet need. Given that the bulk of neuronal loss is pre symptomatic, screenable biomarkers derived from investigations of vesicular proteins may form a basis for opportunities for early treatment with existing therapies and the opportunity to stem novel treatments (Miller and O’Callaghan, 2015).

Another important issue to consider is whether the interactions occur in glial cells vs. neuronal cell types, which may be more relevant for LRRK2 as it is highly expressed in immune cells (Kia et al., 2019). It will be important to identify key facilitators and aggravators of PD (Johnson et al., 2019) in order to develop treatment for patients at the prodromal stage. It will also be critical to tease out the granular details of vesicular pathway disruption at a cellular level, and to distinguish between primary events and secondary sequalae. This will, in turn, provide the foundations for continuing drug development efforts for Parkinson’s.

## Author Contributions

KE made the first draft of the manuscript. PL and RB provided the expert comments and suggestions, and wrote parts of the review. All authors approved the final version of the manuscript.

## Conflict of Interest

The authors declare that the research was conducted in the absence of any commercial or financial relationships that could be construed as a potential conflict of interest.
